# Incidentally Detected Ductal Adenocarcinoma Presenting With Isolated Pulmonary Metastasis and a BRCA2 Mutation

**DOI:** 10.1002/iju5.70103

**Published:** 2025-11-10

**Authors:** Hiroyuki Karasawa, Takeo Kosaka, Akari Komatsuda, Yuto Baba, Kohei Nakamura, Hiroshi Nishihara, Mototsugu Oya

**Affiliations:** ^1^ Department of Urology Keio University School of Medicine Tokyo Japan; ^2^ Department of Urology Kawasaki Municipal Hospital Kanagawa Japan; ^3^ Genomics Unit, Keio Cancer Center Keio University School of Medicine Tokyo Japan

**Keywords:** BRCA2 mutation, comprehensive genomic profiling, ductal adenocarcinoma, PARP inhibitor, prostate cancer

## Abstract

**Background:**

Ductal adenocarcinoma of the prostate is a rare and aggressive cancer that often presents with low prostate‐specific antigen levels and nonspecific urinary symptoms, leading to a delayed diagnosis. Mutations in DNA repair genes such as BRCA2 may render these cancers susceptible to targeted treatments.

**Case Presentation:**

A 78‐year‐old man developed acute urinary retention despite a prostate‐specific antigen of 0.731 ng/mL. Histopathological examination after holmium laser enucleation of the prostate revealed a ductal adenocarcinoma. Imaging revealed multiple lung nodules, and partial lung resection confirmed metastasis. Genomic profiling of the lung metastases revealed a pathogenic BRCA2 mutation. The patient received androgen deprivation therapy, followed by a combination of talazoparib and enzalutamide. Six months later, imaging revealed fewer lung metastases.

**Conclusion:**

This case illustrates the diagnostic challenges of low‐prostate‐specific antigen ductal adenocarcinoma and demonstrates the potential of molecular profiling to guide personalized treatment with targeted therapies for rare prostate cancer subtypes.

**Summary**

- This case illustrates the diagnostic complexity of ductal adenocarcinoma (DAC) and represents the first report of a BRCA2‐mutant DAC treated with talazoparib, highlighting the importance of early molecular testing in guiding personalized therapy for rare prostate cancer subtypes.

**Abbreviations**
ADT: androgen deprivation therapy
CT: computed tomography
DAC: ductal adenocarcinoma
HoLEP: holmium laser enucleation of the prostate
HRR: homologous recombination repair
PSA: prostate‐specific antigen

## 1 Background

Ductal adenocarcinoma (DAC) is a rare histological subtype of prostate cancer. The pure type accounts for 0.1%–0.8% of all prostate malignancies [1], whereas the mixed type with an acinar adenocarcinoma component represents 5.0%–12.7% of radical prostatectomy specimens [2, 3]. Compared to the more common acinar adenocarcinoma, DAC is characterized by a higher histological grade, a greater likelihood of early metastasis, and worse clinical outcomes. Typically occurring in the periurethral region, DAC often presents with obstructive urinary symptoms, such as hematuria or urinary retention. Notably, serum prostate‐specific antigen (PSA) levels may remain within the normal range, even in advanced cases [4], making diagnosis particularly challenging.

Recent advances in molecular oncology have revealed that a subset of prostate cancers, including DAC, harbor alterations in homologous recombination repair (HRR) genes [5, 6]. These alterations confer vulnerability to PARP inhibitors. Although some studies have investigated the genetic profile of the DAC genotype [7], there are few clinical reports of DAC with actionable genomic alterations, and its therapeutic implications remain underexplored.

## 2 Case Presentation

A 73‐year‐old man had been receiving alpha‐blocker therapy for several years to manage lower urinary tract symptoms. On initial transabdominal ultrasound, the prostate volume was estimated to be 53.4 mL. Despite consistently normal PSA levels, the patient developed acute urinary retention at the age of 78 years. The patient's PSA level was 0.731 ng/mL, and a digital rectal examination was not performed since prostate cancer was not suspected. The patient underwent holmium laser enucleation of the prostate (HoLEP) to relieve the obstruction.

**FIGURE 1:**
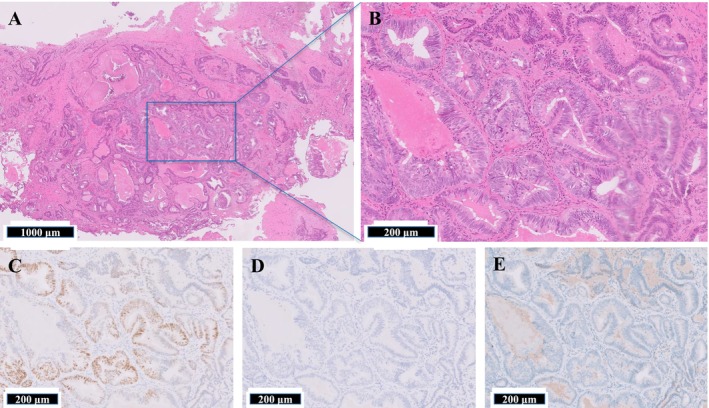
Histological and immunohistochemical analyses of a transurethral resection specimen. (A) Low‐power view of H&E staining shows crowded, irregular glandular structures within the prostatic stroma. (B) High‐power view of H&E reveals cells with prominent nucleoli, nuclear atypia, and frequent mitotic figures, characteristic of ductal adenocarcinoma. (C) Immunostaining for NKX3.1 shows strong nuclear positivity in tumor cells, confirming prostatic origin. (D) Immunostaining for PSA shows negative expression in tumor cells. (E) p40 immunostaining is negative, indicating the absence of basal cells and supporting the diagnosis of invasive carcinoma.

Histologically, the tumor exhibited crowded and architecturally complex glandular structures with marked nuclear atypia and frequent mitotic figures upon hematoxylin and eosin (H&E) staining (Figure 1A,B). Immunohistochemical analysis demonstrated strong nuclear positivity for NKX3.1, confirming the prostatic origin of the tumor, whereas PSA expression was negative (Figure 1C,D). The basal cell marker, p40, was negative, indicating the absence of basal cells and supporting the diagnosis of invasive ductal adenocarcinoma (Figure 1E). These histological and immunophenotypic features were consistent with those of DAC.

**FIGURE 2:**
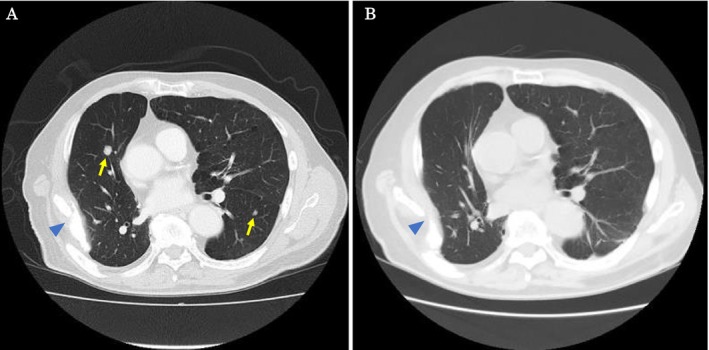
Multiple pulmonary metastases of DAC. (A) Multiple pulmonary nodules of the lung (→). (B) A reduction in the pulmonary metastases following the initiation of ADT and combination therapy with talazoparib and enzalutamide 6 months after diagnosis. ►The patient had previously undergone a right lobectomy with chest wall resection for primary cancer.

Staging computed tomography (CT) revealed multiple pulmonary nodules, raising the differential of primary lung cancer versus metastases (Figure 2A). To confirm the diagnosis, partial resection of the left lower lobe was performed, which demonstrated metastatic DAC. The patient was subsequently referred to our institution, where androgen deprivation therapy (ADT) with degarelix was administered as the initial therapeutic approach. Two months later, recurrent urinary retention occurred, indicating clinical progression despite castration. Given the negative PSA expression in tumor specimens, PSA was unreliable, and this clinical deterioration was regarded as castration resistance.

Because of the atypical clinical presentation and aggressive disease course, comprehensive genomic profiling was performed on the pulmonary metastasis using the FoundationOne CDx (F1CDx) platform. Analysis revealed a pathogenic BRCA2 mutation (Table 1). Based on these results, a combination regimen of talazoparib, a poly (ADP‐ribose) polymerase (PARP) inhibitor, and enzalutamide, an androgen receptor antagonist, was introduced. Six months after diagnosis, CT showed a reduction in pulmonary metastases (Figure 2B).

**TABLE 1:** Genomic analysis summary.

Category	Findings
TMB	4.83 mutations/Mb
MSI status	Stable
HRD signature	Positive
Druggable alterations	PIK3CA p.E545K BRCA2 p.S353 LOH indicated
Germline variant	BRCA2 p.S353 VAF: 54.5%

Abbreviations: HRD, homologous recombination deficiency; LOH, loss of heterozygosity; MSI, microsatellite instability; TMB, tumor mutational burden; VAF, variant allele frequency.

## 3 Discussion

In the present case, the patient was managed for benign prostatic hyperplasia until the development of urinary retention. Despite a low PSA level, HoLEP revealed underlying DAC. PSA levels may remain low even in advanced cases, which underscores the diagnostic value of specimens obtained during nonmalignant procedures.

Staging investigations identified isolated pulmonary metastases, and a diagnosis of metastatic DAC was histologically confirmed after partial resection of the lobe. Patients with DAC have metastases at diagnosis at a three‐times higher rate than patients with acinar adenocarcinoma [8]. Although the most common sites of metastases are bone and lymph node, which are similar to acinar adenocarcinoma, DAC tends to spread to visceral organs such as the lungs and brain [9]. In a review of metastatic patterns, lung metastases in DAC occur in 23.3% of cases at diagnosis, often in conjunction with other metastatic sites [10]. Purely pulmonary involvement, as observed here, remains rare and may suggest unique tumor biology or molecular drivers.

This led to a key finding in this case: the identification of a BRCA2 mutation in the metastatic lung lesion via F1CDx. Mutations in BRCA1/2, a crucial component of the HRR pathway, are increasingly recognized as biomarkers of aggressive disease and treatment responsiveness in prostate cancer. Alterations in the HRR pathway are frequently observed in patients with DAC, with one study reporting HRR mutations in 31% of cases [5]. BRCA1/2 mutations are associated with higher Gleason scores, nodal involvement, and metastasis at diagnosis [11]. Furthermore, BRCA2 alterations are associated with poorer outcomes [12], underscoring the need for early molecular testing.

Importantly, BRCA2 mutations confer sensitivity to PARP inhibitors, a class of agents that target cells with deficient DNA damage repair. The TALAPRO‐2 trial recently demonstrated that the addition of the PARP inhibitor talazoparib to enzalutamide significantly improved radiographic progression‐free survival (rPFS) in patients with metastatic castration‐resistant prostate cancer and HRR mutations [13]. This finding supports the rationale for early use of PARP inhibitor‐based combination therapy in BRCA2‐mutated mCRPC.

In the updated analysis of TALAPRO‐2, the median rPFS with talazoparib plus enzalutamide was 33.1 months (95% CI 27.4–39.0), providing an estimate of the expected timeframe of disease control; however, the durability of benefit in DAC remains to be clarified [13]. Because PSA levels are often unreliable for monitoring disease activity in DAC, careful assessment of clinical symptoms and radiographic findings is essential for evaluating treatment response and progression.

This case also underscores the utility of performing comprehensive genomic profiling (CGP) of metastatic tissues, particularly in rare histological subtypes such as DAC. The use of CGP platforms, such as F1CDx, allows the identification of actionable mutations, which in turn can inform personalized treatment strategies, even at earlier disease stages.

This case highlights the importance of integrating advanced imaging, histopathological findings, and molecular diagnostics for the management of atypical prostate cancers. For patients with rare subtypes, such as DAC, especially those with visceral metastases and low PSA levels, early genomic testing should be considered to guide therapeutic decisions and optimize outcomes.

## 4 Conclusion

To the best of our knowledge, this is the first case report on the use of talazoparib to treat DAC with a BRCA2 mutation. This case illustrates the diagnostic complexity and aggressive nature of low‐PSA DAC, incidentally detected following HoLEP. The discovery of a pathogenic BRCA2 mutation enabled the consideration of PARP inhibitor‐based combination therapy, emphasizing the critical role of molecular diagnostics in guiding individualized treatment for rare prostate cancer subtypes.

## Consent

All informed consent was obtained from the patient.

## Conflicts of Interest

The authors declare no conflicts of interest.
